# Insights into the Evolution of IncR Plasmids Found in the Southern European Clone of the Monophasic Variant of *Salmonella enterica* Serovar Typhimurium

**DOI:** 10.3390/antibiotics13040314

**Published:** 2024-03-29

**Authors:** Xenia Vázquez, Javier Fernández, Jürgen J. Heinisch, Rosaura Rodicio, M. Rosario Rodicio

**Affiliations:** 1 Departamento de Biología Funcional, Área de Microbiología, Universidad de Oviedo (UO), 33006 Oviedo, Spain; xenia.vazquez@ipla.csic.es (X.V.); fernandezdjavier@uniovi.es (J.F.); 2 Grupo de Microbiología Traslacional, Instituto de Investigación Sanitaria del Principado de Asturias (ISPA), 33011 Oviedo, Spain; mrosaura@uniovi.es; 3 Servicio de Microbiología, Hospital Universitario Central de Asturias (HUCA), 33011 Oviedo, Spain; 4 Centro de Investigación Biomédica en Red-Enfermedades Respiratorias, 30627 Madrid, Spain; 5 Research & Innovation, Artificial Intelligence and Statistical Department, Pragmatech AI Solutions, 33001 Oviedo, Spain; 6 Department of Genetics, Faculty of Biology and Chemistry, University of Osnabrück, Barbarastrasse 11, D-49076 Osnabrück, Germany; jheinisc@uni-osnabrueck.de; 7 Departamento de Bioquímica y Biología Molecular, Universidad de Oviedo (UO), 33006 Oviedo, Spain

**Keywords:** monophasic variant of Typhimurium, Southern European clone, ST19, multidrug resistance, IncR, resistance-virulence plasmid, plasmid evolution, phylogenetic analysis

## Abstract

*Salmonella enterica* subspecies *enterica* serovar 4,[5],12:i:- is a monophasic variant of *S*. Typhimurium which has emerged as a world-wide distributed pathogen in the last decades. Several clones have been identified within this variant, the European clone, the Spanish clone, the Southern European clone and the U.S./American clone. The present study focused on isolates of the Southern European clone that were obtained from clinical samples at Spanish hospitals. The selected isolates were multidrug resistant, with most resistance genes residing on IncR plasmids that also carried virulence genes. These plasmids had a mosaic structure, comprising a highly reduced IncR backbone, which has acquired a large amount of exogenous DNA mostly derived from pSLT and IncI1-I(alfa) plasmids. Although composed of approximately the same elements, the investigated plasmids displayed a high diversity, consistent with active evolution driven by a wealth of mobile genetic elements. They comprise multiple intact or truncated insertion sequences, transposons, pseudo-compound transposons and integrons. Particularly relevant was the role of IS*26* (with six to nine copies per plasmid) in generating insertions, deletions and inversions, with many of the rearrangements uncovered by tracking the patterns of eight bp target site duplications. Most of the resistance genes detected in the analyzed isolates have been previously associated with the Southern European clone. However, *erm*(B), *lnu*(G) and *bla*_TEM-1B_ are novel, with the last two carried by a second resistance plasmid found in one of the IncR-positive isolates. Thus, evolution of resistance in the Southern European clone is not only mediated by diversification of the IncR plasmids, but also through acquisition of additional plasmids. All isolates investigated in the present study have the large deletion affecting the *fljBA* region previously found to justify the monophasic phenotype in the Southern European and U.S./American clones. An SNP-based phylogenetic analysis revealed the close relationship amongst our isolates, and support that those sharing the large *fljBA* deletion could be more heterogeneous than previously anticipated.

## 1. Introduction

Non-typhoid serovars of *Salmonella enterica* are one of the major causes of bacterial food-borne gastrointestinal infections worldwide [1,2]. More than 2600 serovars have been identified in this complex species [3], but three of them, *S*. Enteritidis, *S*. Typhimurium and *S*. 4,[5],12:i:-, are by far the most commonly involved in clinical human infections in many parts of the world, including Europe [1].

In *S. enterica*, two flagellar phases can be alternatively expressed. Phase variation relies on the switch of transcription between the *fliC* and *fljBA* genes, which encode the phase 1 flagellin (*fliC*), the phase 2 flagellin (*fljB*), and a transcriptional repressor of *fliC* (*fljA*). The switch between *fliC* and *fljB* expression is mediated by site specific inversion of a DNA segment, named segment H, catalyzed by a DNA invertase encoded by the *hin* gene. Depending on the orientation of this segment, which is flanked by the *hixL* and *hixR* recombination sites and contains the promoter of the *fljBA* genes, either *fliC* or *fljBA* are expressed [4]. Although considered as an independent serovar for epidemiological surveillance, several lines of evidence support that *S*. 4,[5],12:i:- is a monophasic variant of *S*. Typhimurium which lacks the second phase flagellar antigens, represented by 1,2 in the antigenic formula of biphasic *S*. Typhimurium (4,[5],12:i:1,2). This is supported by subtyping results, including phage typing, PFGE (Pulsed-Field Gel Electrophoresis) and MLST (MultiLocus Sequence Typing), and by phylogenetic analysis [5,6,7,8,9,10].

Since the 1990s, this monophasic serovar has been increasingly detected, currently representing a public health hazard worldwide [7,8,9,11,12,13,14,15]. However, it soon became clear that *S*. 4,[5],12:i:- is a heterogeneous serovar, comprising multiple clonal lines which emerged at different geographical locations through independent deletion events affecting the *hin*-*fljBA* chromosomal region of diverse *S*. Typhimurium ancestors [16]. The predominant clone, known as the European clone, displays multidrug resistance (MDR) encoded by chromosomal genes, and belongs to sequence type (ST) 34. First reported in Europe, this clone has spread all over the world, with its proportion increasing constantly since around 2005 [5,7,15,17]. Other *S*. 4,[5],12:i:- clones, including the originally reported Spanish clone, the Southern European clone and the U.S./American clone, belong to ST19. Isolates of the U.S./American clone are mostly susceptible to antibiotics [16,18], while those pertaining to both the Spanish and the Southern European clones are MDR, and their resistance properties are conferred by plasmids of the IncC (formerly known as IncA/C [19]) and the IncR incompatibility groups, respectively [20,21,22,23,24,25,26]. Interestingly, both types of plasmids also contain virulence genes derived from pSLT, the virulence plasmid specific for *S*. Typhimurium [21,22,23,26].

The term “Southern European clone” was coined to accommodate monophasic isolates obtained from human clinical samples, food of animal origin and the environment in Portugal which shared the same chromosomal deletion removing the *fljBA* genes, as the U.S. isolates. However, they were resistant to chloramphenicol, streptomycin, sulfamethoxazole, tetracycline and trimethoprim due to the acquisition of IncR plasmids [25]. Similar isolates were also collected in Italy and Spain from human and swine samples, and their IncR plasmids carried pSLT-virulence genes together with the resistance genes [21,23].

Given the high incidence and global distribution of the European ST34 clone, it has been extensively studied. On the other hand, information on the monophasic ST19 clones is comparatively scarce, particularly for the Southern European clone (reviewed in [8]). For instance, the sequence of only a single IncR plasmid (pST1023) carried by an Italian isolate belonging to the latter clone has been reported so far [21]. Taking this into account, we performed detailed conventional and genomic analyses of six isolates pertaining to the latter clone, which were obtained from human clinical samples in Spain between 2012 and 2018. Special attention was paid to the diversity and evolution of the IncR plasmids which confer resistance and virulence properties to the Southern European monophasic clone.

## 2. Results

### 2.1. Origin and Typing of the Isolates Belonging to the Southern European Clone

The six isolates of the Southern European clone selected for the present study derived from the collection of *S.* 4,[5],12:i:- clinical isolates recorded at the LSP (Laboratorio de Salud Pública, Asturias, Spain) in the years 2012–2018. They were obtained from human feces (5) or blood (1) at three different hospitals in our region (Table 1). According to information provided by the CNM, four and two isolates presented the antigenic formula 4,5,12:i:- and 4,12:i:-, respectively, and in silico serotyping supported the monophasic phenotype. Regarding phage type, and also according to CNM information, the isolates were DT (Definitive Type) 18 (2), DT104 (1), DT120 (1) or RDNC (Reacted but Did Not Conform; 2). As expected for the Southern European clone, the sequence type was ST19 as determined by MLST performed in silico.

### 2.2. Patterns of Antibiotic Resistance and Identification of the Responsible Genes

Overall, resistances to ampicillin (encoded by *bla*_TEM-1B_), chloramphenicol (*cmlA1*), streptomycin (*aadA1*, *aadA2*, *aadA22*, Δ*strA* and *strB*), sulfonamides (*sul3*), tetracycline [*tet*(B)] and trimethoprim (*dfrA12*) were detected in the analyzed isolates. By combining the resistance phenotypes and genotypes, four resistance profiles, termed R1 to R4, were identified. The most frequent profile was R1, shared by three isolates, while each of the other profiles was associated with a single isolate. ResFinder correctly identified all resistance genes previously detected by PCR amplification. Moreover, bioinformatics analyses detected the presence of the *erm*(B) gene, associated with the MLS (macrolide-lincosamide-streptogramin B) phenotype, and the *lnu*(G) gene, for lincosamide resistance, each in a single isolate. However, all isolates were susceptible to azithromycin while lincosamides susceptibility was not tested (see Section 4). Moreover, the *silESRCFBAGP* cluster for silver resistance was found in two isolates, the *merR* and *merT* genes in four isolates, and the *qacH* gene for resistance quaternary ammonium compounds in all of them (Table 1). The MIC of AgNO_3_ of the two isolates positive for the *sil* genes (LSP 40/12 and LSP 40/13) was 62 µM, twice the value obtained for the isolates of the Southern European clone lacking the *sil* cluster, and that for *S.* Typhimurium LT2, included as negative control (31 µM). The MIC of the positive control, LSP 389/97, belonging to the monophasic Spanish clone was considerably higher (125 µM) [26].

### 2.3. Plasmid Analysis

The presence of plasmids was experimentally investigated by PBRT and complemented with in silico analysis of the sequenced genomes. According to their assignment to the Southern European clone, a large IncR plasmid was detected in each isolate, and a second resistance plasmid, of unidentified incompatibility group, was found in a single isolate (LSP 40/13). In addition, small cryptic plasmids (of less than ca. 4.6 kb) belonging to the ColE group or with an unknown replicon were observed in two out of the six isolates (Table 1).

#### 2.3.1. Comparative Analysis of the IncR Plasmids and Phylogenetic Relationships

**Figure 1:**
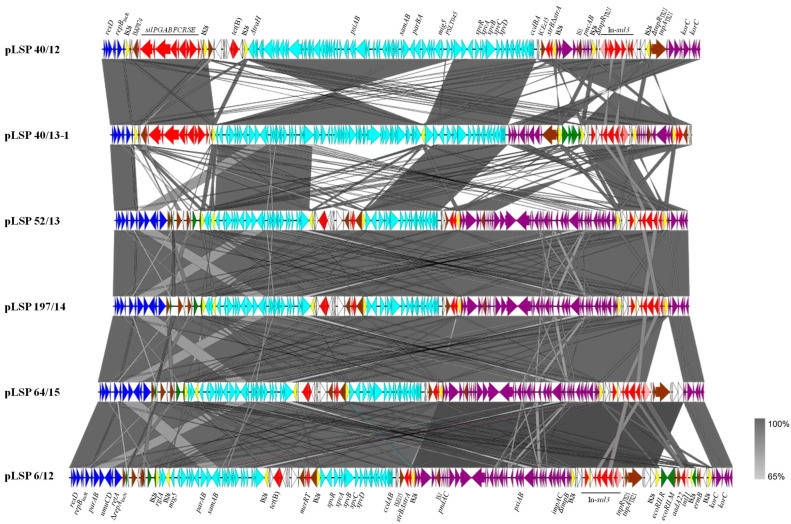
Comparison of the reconstructed IncR plasmids found in Spanish isolates of the Southern European monophasic clone. Coding regions are represented by arrows indicating the direction of transcription and colored according to their origin or function: dark blue, IncR backbone; pale blue, pSLT DNA; purple, IncI1-I(alfa) DNA; green, DNA traced to plasmids of other incompatibility groups; red, resistance genes; yellow, IS*26*; brown, genes from other transposable elements, including insertion sequences and transposons; pink, *intI1* gene encoding the integrase of class 1 integrons; white, orfs of unknown origin. Large but incomplete sets of genes involved in conjugation are carried by the pSLT and IncI1-I(alfa) segments, although they are not specified in the Figure. The alignments were created with EasyFig 2.2.5 blastn. The gray shading between regions reflects nucleotide sequence identities according to the scale shown at the right lower corner of the figure.

Figure 1 shows a comparison of the IncR plasmids reconstructed in the present study, whose sizes ranged between ca. 119 and 138 kb. Based on the length of the IncR backbone, two groups could be established, in which it accounted for only approximately 5 or 11 kb, respectively. Both contain the *repB* gene responsible for initiation of replication, a set of iterons controlling *repB* expression and therefore copy number, and a *resD*-like gene coding for a resolvase, probably involved in multimer resolution. In four out of the six plasmids, the IncR backbone also contained the *parA* and *parB* genes for accurate plasmid partition, the *umuC* and *umuD* genes involved in DNA repair and the *retA* gene, encoding a group IIB intron reverse transcriptase. The remaining DNA could be mostly traced to pSLT, the virulence plasmid specific for *S*. Typhimurium (IncFII_S_ + IncFIB), and to IncI1-I(alpha) plasmids. Small segments derived from IncP, IncN and IncFIB plasmids were also found in some but not all plasmids. Within the exogenous DNA, multiple resistance genes were associated with a wealth of intact or truncated mobile genetic elements, including integrons, insertion sequences, transposons and pseudo-compound transposons (PCTs), i.e., structures bounded by directly-oriented copies of IS*26* [27]. Particularly relevant is the presence of six, seven and nine copies of this insertion sequence (which belongs to the IS*6* family) in two, three and one of the IncR plasmids, respectively (Figure 1).

pLSP 40/12 and pLSP 40/13-1 are the two plasmids with the shortest IncR backbone. Downstream of this highly reduced backbone, both contained the *sil* region, responsible for enhanced silver resistance (Section 2.2). This region, which also encompasses insertion sequence IS*Kpn74* (IS*5* family), is flanked by two copies of IS*26* in the same orientation. Further downstream, and preceded by another copy of IS*26*, appears the pSLT DNA that in pLSP 40/12 consists of a large continuous segment of ca. 59.4 kb that spans from Δ*traH* to *ccdA*. Within this segment, the genes *psiAB* (inhibition of plasmid SOS), *samAB* (homologous to *umuDC* [28]), *parBA*_pSLT_ for plasmid partition, *ccdBA* encoding a toxin-antitoxin system, and a large but incomplete set of genes involved in conjugation are found. The *spv* (*Salmonella* plasmid virulence) locus and the virulence gene *mig5* (macrophage-inducible gene [29]) are also placed within this segment (see Section 4). In pLSP 40/13-1, the pSLT DNA is subdivided into two blocks, separated by an additional copy of IS*26*. The first block (42.7 kb) spans between ΔPSLT045 (following the annotation used in NC_003277; 5′-end) and Δ*traH* and is inverted with respect to the equivalent region found in pLSP 40/12 and flanked by oppositely-oriented copies of IS*26*. In agreement with the IS*26*-mediated inversion of the segment, the location and orientation of the TSDs (target site duplications) are altered, appearing in opposite orientation at the conventional 3′-end of the two IS (iTSD: CGATATAC/TSD: GTATATCG, originally present in PSLT045). The second block (16.6 kb) extends between ΔPSLT045 (3′-end) and *ccdA*. Except for PSLT045, which is intact in pSLP 40/12 and disrupted in pLSP 40/13-1, all other pSLT genes coincide in the two plasmids. Finally, a defective Tn*10* transposon carrying *tet*(B) for tetracycline resistance is located between the *sil* region and the pSLT DNA in pLSP 40/12 but not in pLSP 40/13-1, which belongs to the only isolate susceptible to tetracycline (Table 1). Like the *sil* region of the two plasmids, the *tet*(B) region of pLSP 40/12 is flanked by equally-oriented copies of IS*26*, giving rise to PCTs.

The remaining DNA is very similar in pLSP 40/12 and pLSP 40/13-1, but occurs in opposite orientations. It consists of two segments that could be tracked to plasmids of the IncI1-I(alpha) group, and two resistance regions. One of the latter contains the Δ*strA* and *strB* genes bounded by IS*Ecl5* (IS*3* family) and IS*26*, while the other consists of a *sul3*-class 1 integron with the *intI1*/*dfrA12*-*orfF*-*aadA2*-*cmlA1*-*aadA1*-*qacH*-IS*440*-*sul3* configuration, assigned to type I [30]. The first IncI1 I(alfa) segment (ca. 7.4 kb) comprises genes involved in plasmid stability (*pndAC* coding for a toxin-antitoxin system) and another defective set of genes for conjugational transfer. This segment is more closely related to DNA present in several IncI1-I(alfa) plasmids from *S. enterica* (accession numbers CP053578, KX058576 and CP016520) and from multiple strains of *E. coli*. However, they differ by the presence of IS*1* inserted within the IncI1-I(alfa) DNA of our plasmids but not in the homologous regions of other plasmids. The second IncI1-I(alfa) segment, of ca. 10.5 kb, is located downstream or upstream of the *sul3*-type I integron in pLSP 40/12 and LSP 40/13-1, respectively. It includes two *korC*-like regulatory genes and is identical to a region found in pB39-I, an *E. coli* plasmid of the IncI1-I(alpha) group, which also carries the *sul3*-type 1 integron (accession number OQ420470). Upstream of the integron, an additional region (of ca. 3.8 kb; bounded by directly oriented IS*26*) is found in pLSP 40/13-1 but not in pLSP 40/12. This region is nearly identical to a region from plasmids of the IncP incompatibility group found in different *Enterobacterales* (i.e., CP035316). Both in pLSP 40/12 and pLSP 40/13-1, the *sul3*-type I integron is flanked by oppositely-oriented IS*26*, associated with iTSD (GTCGCCGG) and TSD (CCGGCGAC; belonging to the *tnpR* gene coding for the resolvase of Tn*21*). In pLSP 40/13-1, the segment also encompasses the IncP DNA.

All other IncR plasmids analyzed in the present study (pLSP 6/12, pLSP 52/13, pLSP 197/14 and pLSP 64/15; Figure 1) belong to the second group. They contain the larger IncR backbone and lack the *sil* genes, consistent with the silver susceptibility of the isolates in which they were detected (Section 2.2). Instead, following the boundary of the IncR backbone, there is a complex region which comprises a truncated *repA* gene of the IncN incompatibility group, a small segment derived from IncFIB plasmids, and multiple insertion sequences belonging to two different families: IS*1X3* and IS*1X2* (IS*1* family) and IS*903B* (IS*5* family). Downstream of this region, they contain two pSLT segments, with the first inverted with respect to the homologous region in pSLT and flanked by oppositely-oriented copies of IS*26*, as previously shown for pLSP 40/13-1. However, the two segments do not exactly coincide with each other and in plasmids of the second group they are separated by the *tet*(B)-containing element which has the *merRT* genes at the 3′-end. Moreover, all plasmids of the second group carry an extra copy of IS*26* inserted within the first pSLT segment, in the intergenic region between ΔPSLT044 (annotated as *rglA* in the pSLT sequence but coinciding with the *tnpA* gene of an insertion sequence of the IS*481* family) and *mig5*. In pLSP 52/13 and pLSP 197/14 (which are nearly identical to each other), IS*26* is delineated by eight bp TSDs (GTGAAAAC). pLSP 6/12 and pLSP 64/15 have the small segment inverted and flanked by oppositely oriented copies of IS*26*, associated with iTSD (GTTTTCAC) and TSD (GTGAAAAC).

The remaining DNA of these plasmids includes the *strB*-Δ*strA* genes and two segments derived from IncI1-I(alpha) plasmids separated by the *sul3*-type I-integron, as previously observed for pLSP 40/12 and pLSP 40/13-1. However, the first IncI1-I(alfa) segment is much bigger (32.5 kb vs. 7.4 kb), including a larger set of conjugative genes together with genes involved in plasmid stability (*pndAC*), SOS response inhibition (*psiAB*) and translesion error-prone repair (*impCA*-Δ*impB*), among others. Interestingly, in one of the plasmids (pLSP 6/12), an additional region of ca. 11.7 kb was inserted within the second IncI1-I(alfa) segment. The additional DNA comprises the genes encoding the EcoRII restriction–modification system, the *intI1*/*aadA22* genes of a second class 1 integron, and the *erm*(B) gene, first reported in the Southern European clone. The entire region is delineated by equally-oriented copies of IS*26* flanked by eight bp TSDs (TGCTGGAC), which belong to an IncI1-I(alpha) gene of unknown function. Another copy of IS*26* appears between the defective class 1 integron and the *erm*(B) gene. Nearly identical DNA sequences are carried by pR17.4111_p113k, a plasmid of *S.* Enteritidis (CP063290), also belonging to the IncI1-I(alfa) group.

#### 2.3.2. The Second Resistance Plasmid of LSP 40/13

**Figure 2:**
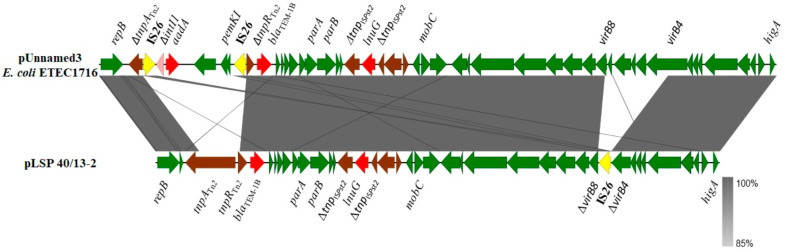
Comparison of an *lnu*(G)-containing plasmid carried by a Spanish isolate of the Southern European monophasic clone with a related plasmid found in *Escherichia coli* strain ETEC1716 (accession number CP122883). Coding regions are represented by arrows indicating the direction of transcription and colored according to their origin or function: red, resistance genes; yellow, IS*26*: brown, genes from other transposable elements, including insertion sequences and transposons; pink, *intI1* gene; green, all other plasmid genes. Apart from *virB8* and *virB4* (truncated in pLSP 40/13-2), other genes involved in conjugationare carried by both plasmids, but they are not specified. The alignments were created with EasyFig 2.2.5 blastn. The gray shading between regions reflects nucleotide sequence identities according to the scale shown at the right lower corner of the figure.

Although most of the resistance properties displayed by the isolates of the Southern European clone were conferred by IncR plasmids, two resistance genes, *bla*_TEM-1B_ and *lun*(G), were carried by pLSP 40/13-2, a second plasmid detected in LSP 40/13 (Figure 2). pLSP 40/13-2 comprises 33.9 kb and belongs to a yet unknown incompatibility group. Within this plasmid, the *lnu*(G) gene is inserted into the transposase gene of IS*Pst2*, an insertion sequence of the IS*L3* family first reported in *Pseudomonas stutzeri* strain OX1 [31]. The insertion sequence is flanked by eight bp TSDs (GATTTATC), which belong to the intergenic region between two genes encoding hypothetical proteins and located downstream and upstream of *parB* and *mobC* of pLSP 40/13-2, respectively. This plasmid also contains the *bla*_TEM-1B_ gene as part of transposon Tn*2*. Neither *lnu*(G) nor *bla*_TEM-1B_ were previously found in the Southern European monophasic clone.

According to blastn comparisons, plasmids related to pLSP 40/13-2, sharing more than 97% identity with a 68–92% coverage, were only found in several *Enterobacterales*, including two strains of *S. enterica*, one of serovar *S.* Enteritidis and another of unidentified serovar, as well as in several isolates belonging to *Escherichia coli*, *Klebsiella pneumoniae*, *Citrobacter*, *Serratia* and *Erwinia amylovora*. Only two of these plasmids, one from the *E. coli* enterotoxigenic strain ETEC1712 (accession number CP122883) and the other from *C. braakii* (CP126334), carried *lnu*(G). A comparison of pLSP40/13-2 with the *E. coli* plasmid is shown in Figure 2. They only differ by the presence of an IS*26* inserted within the *vir* region of pLSP 40/13-2, and of an additional element carrying a defective Δ*int*/*aadA* integron and the *pemK*-*pemI* antitoxin-toxin genes in the *E. coli* plasmid. This element is flanked by oppositely-oriented copies of IS*26* and has integrated into the Tn*2* transposon which harbours *bla*_TEM-1B_.

### 2.4. Genetic Basis of the Monophasic Phenotype and Phylogenetic Analysis

**Figure 3:**
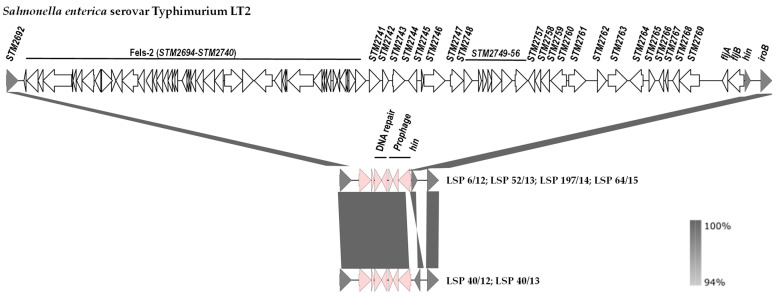
Comparison of the DNA regions located between STM2692 and *iroB* in the chromosomes of *Salmonella enterica* serovar Typhimurium LT2 (genes are named according to accession number AE006468.1) and the isolates of the Southern European clone analyzed in the present study. The alignments were created with EasyFig 2.2.5 blastn. The gray shading between regions reflects nucleotide sequence identities according to the scale shown at the right lower corner of the figure. Genes are represented as arrows pointing in the direction of transcription. Color code: grey, genes present in *S*. Typhimurium LT2 and the isolates of the Southern European clone; white, genes present only in *S*. Typhimurium LT2; pink, genes present in isolates of the Southern European clone but not in the equivalent chromosomal region of *S*. Typhimurium LT2. Please note that the *hin* gene responsible for phase variation is oppositely oriented in 4 and 2 isolates of the Southern European clone.

The genetic basis of the monophasic phenotype was established by comparison of the *hin*-*fljBA* chromosomal region of *S.* Typhimurium LT2 with the corresponding regions of the isolates under study (Figure 3). Coinciding with information previously reported for the Southern European and also for the U.S/American clone [20,25], our isolates had a large deletion of ca. 70.5 kb, which starts downstream of STM2692 and ends within the *hixL* inverted repeat of segment H, retaining the *hin* gene that encodes the invertase responsible for phase variation in the biphasic isolates (Figure 3).

The deletion removes the *fljA* and *fljB* genes, thereby accounting for the monophasic phenotype, together with the prophage Fels-2 genome, and the STM2741 to STM2769 region. In all cases, the missing DNA was replaced by a ca. 5.3 kb bp insert, which contains prophage genes, genes involved in DNA repair (*umuC* and another one encoding an SOS response-associated peptidase) or non-annotated orfs. It is noticeable that the two isolates of the first group (LSP 40/12 and LSP 40/13) and four isolates of the second group (LSP 6/12, LSP 52/13, LSP 197/14 and LSP 64/15) carried segment H in opposite orientations.

Finally, to investigate the relationships between the isolates under study, an SNP-based phylogenetic tree was constructed, also including the genomes of the Italian ST1023 isolate and of 25 other isolates containing the large *fljBA* deletion responsible for the monophasic phenotype in the Southern European and the U.S./American clones. All isolates selected in this way were ST19, except one which was ST7910 (a single locus variant of ST19 having the *sucA*_1113 allele instead of *sucA*_9). According to the available information, most of them were collected in American countries (U.S, Canada and Trinidad and Tobago), but some originated in Asia (South Corea and China). They derived from different sources, mainly human clinical samples, and to a lesser extent from food-producing animals, food and the environment (Table S2).

**Figure 4:**
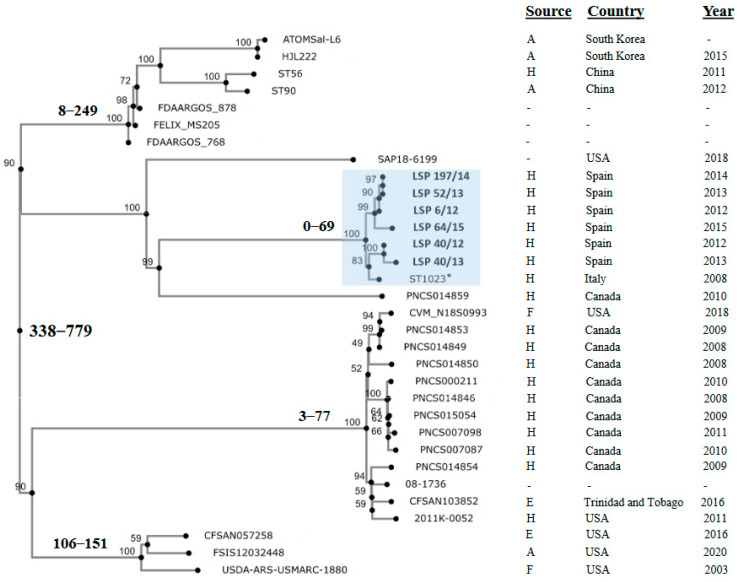
Phylogenetic position of Spanish isolates belonging to the Southern European monophasic clone in the context of isolates from different countries, which shared the same deletion responsible of the monophasic phenotype. All selected isolates were ST19 except ST56 from China that was ST7910. The tree was constructed with the CSI Phylogeny 1.4 (https://cge.food.dtu.dk/services/CSIPhylogeny/; last accessed on 25 February 2024) using the genome of LSP 6/12 as the reference for SNP calling. Values at each node represent percent bootstrap support based on 1000 replicates. For relevant nodes, the minimum and maximum SNP are also indicated in bold. The cluster containing the Spanish isolates (shown in bold) and the Italian isolate (marked with an asterisk), all belonging to the Southern European clone, is highlighted in blue. Relevant information related to the isolates is compiled at the right of the figure. Accession numbers of the genomes and the pairwise distance matrix used to construct the phylogenetic tree are shown in Table S2 and Table S3, respectively.

Isolates in the phylogenetic tree differed by a maximum of 779 nt (Figure 4; Table S3). Those analyzed in the present study were separated by 0 (LSP 52/13 vs. LSP 197/14) to 69 (LSP 40/13 vs. LSP 64/15) SNPs, forming a tight clonal group, supported by a 100% bootstrap value, which also included the Italian ST1023 isolate. The latter differed from the Spanish isolates by 39 to 53 SNP. The phylogenetic distances with the remaining isolates were considerably higher, ranging between 510 and 776 SNP. Those isolates were distributed into several other clusters or appeared as independent branches. One of these clusters contained the Asian isolates together with others of unknown provenance, while within the rest, isolates from American countries were grouped.

## 3. Materials and Methods

### 3.1. Isolate Selection

All 603 *S*. 4,[5],12:i:- isolates recovered from human clinical samples between 2012 and 2018 in a Northern Spanish region (Asturias) were experimentally tested for antimicrobial susceptibility, resistance genes and plasmid content (to be published elsewhere). Six isolates that proved to be positive for the IncR replicon and had an R-pattern (phenotype and genotype) consistent with the Southern European clone were selected for the present study. The serotype and phage type were experimentally determined at the CNM (Centro Nacional de Microbiología, Madrid, Spain).

The presence of IncR plasmids was demonstrated by PBRT (PCR-Based Replicon Typing [32]). Antimicrobial susceptibility was determined by disk diffusion assays using Mueller–Hinton agar and commercially available discs (Oxoid, Madrid, Spain). The tested compounds (with the amount per disk in µg given in parentheses) were ampicillin (10), amoxicillin-clavulanic acid (30), cefepime (30), cefotaxime (30), cefoxitin (30), erthapenem (10), chloramphenicol (30), amikacin (30), gentamicin (10), kanamycin (30), streptomycin (10), tobramycin (10), azithromycin (15), nalidixic acid (30), ciprofloxacin (5), sulfonamides (300), tetracycline (30), trimethoprim (5), fosfomycin (300) and nitrofurantoin (300). Results were interpreted according to EUCAST (The European Committee on Antimicrobial Susceptibility Testing; https://eucast.org/clinical_breakpoints/; last accessed on 12 December 2023) or to CLSI (Clinical and Laboratory Standards Institute) guidelines [33]). Genes responsible for resistance to ampicillin [*bla*_TEM-1B_, *bla*_OXA-1_, *bla*_PSE-1_], chloramphenicol [*cmlA1, catA1, floR*], aminoglycosides [*aac(3)-IV, aadA1*-like, *aadA2, strA, strB*], sulfonamides [*sul1, sul2, sul3*], tetracycline [*tet*(B), *tet*(A)] and trimethoprim (*dfrA1*-like, *dfrA12*) were detected by simplex or multiplex PCR-amplifications [34]. The MIC (Minimum Inhibitory Concentration) of silver was determined by microdilution tests using AgNO_3_ at concentrations ranging between 0 and 125 µM. *S*. Typhimurium LT2 and *S*. 4,5,12:i:- LSP 389/97, previously shown to be either susceptible or resistant to silver [26], were used as negative and positive controls, respectively.

### 3.2. Whole Genome Sequencing, Assembly, Annotation and Bioinformatics Analysis

The whole genome sequence of the six IncR-positive isolates was generated with short-read Illumina technology. Genomic DNA was purified from overnight cultures grown in Luria–Bertani broth, using the GenElute^TM^ Bacterial Genomic DNA Kit (Sigma-Aldrich; Merck Life Science, Madrid, Spain). Sequencing was performed in a HiSeq 2500 at the CIBIR (Centro de Investigación Biomédica de La Rioja, Logroño, Spain) or in a NovaSeq 6000 S2 PE150 XP at Eurofins Genomics (Ebersberg, Germany). In both cases, paired-end reads of 90 or 150 nucleotides were obtained from ca. 500 bp fragment libraries. The obtained raw reads were assembled into contigs with the VelvetOptimiser.pl script implemented in the online version of PLACNETw, which also assigns the created contigs to either the chromosome or to plasmids, based on BLAST searches and coverage (https://castillo.dicom.unican.es/upload/; last accessed on 28 April 2021 [35]). The genomes were deposited in GenBank under the accession numbers provided below (see also Table S1, which contains additional information about the assembled genomes), and annotated by the NCBI Prokaryotic Genome Annotation Pipeline (PGAP; https://www.ncbi.nlm.nih.gov/genome/annotation_prok/; last accessed on 18 April 2022 [36]).

Bioinformatics analyses were performed with PLACNETw, in combination with several tools available at the Center for Genomic Epidemiology (CGE) of the Technical University of Denmark (DTU), including SeqSero 1.2, MLST 2.0, ResFinder 4.5.0, PlasmidFinder 2.1 and pMLST 2.0 (https://cge.cbs.dtu.dk/services/; last accessed on 22 March 2024). Insertion sequences were accurately assigned using ISfinder (https://isfinder.biotoul.fr/; last accessed on 14 February 2024). Reconstruction of the resistance plasmids was accomplished by closing the gaps between overlapping contigs of plasmids origin, identified by PLACNETw, using PCR reactions with specific primers pairs (available upon request), followed by Sanger sequencing of the obtained amplicons (performed at Eurofins Genomics), when required. Analysis of *fljBA* regions was achieved with the aid of blastn, CLONE Manager (CloneSuit9), and also with a customized data base comprising all open reading frames (ORF) located between STM2692 and *iroB* in the chromosome of biphasic *S*. Typhimurium LT2 (accession number AE006468.1 [37]). Graphic representations were obtained with EasyFig 2.2.5 (https://mjsull.github.io/Easyfig/; last accessed on 12 February 2024).

### 3.3. Phylogenetic Analysis

An SNP (Single Nucleotide Polymorphism)-based phylogenetic tree was built for the genomes of the six isolates sequenced in the present study, the Italian ST1023 isolate whose IncR plasmid was sequenced [21], and 25 other monophasic isolates potentially belonging to the Southern European or the U.S./American clone. The latter were selected by blastn searches of the insert associated with a large deletion responsible for the monophasic phenotype in the two clones, and the flanking DNA. The origin, country and date of detection of the isolates, as well as the accession numbers of the genomes, which were retrieved from GenBank-NCBI (https://www.ncbi.nlm.nih.gov/genbank/; last accessed on 16 February 2024), are compiled in Table S2. To construct the tree, the CSI phylogeny tool (version 1.4) available at the CGE website [38] was used. The pipeline was run with default parameters, using LSP 6/12 as the reference for SNP calling. The pairwise SNP distance matrix used to generate the tree is shown in Table S3. Bootstrap support for the consensus tree was based on 1000 replicates [39].

## 4. Discussion

In *S. enterica*, two types of “hybrid” plasmids have evolved through acquisition of either resistance genes by serovar-specific virulence plasmids (VR plasmids) or of virulence genes by resistance plasmids belonging to different incompatibility groups (RV plasmids). Both types of such plasmids have been reported in the emergent monophasic 4,[5],12:i:- variant of *S*. Typhimurium [40]. For instance, RV plasmids belonging to the incompatibility groups IncC and IncR were specifically associated with the Spanish and Southern European ST19 clones, respectively [21,22,23,24]. Monophasic isolates positive for RV plasmids of the IncR group were first recovered from human and swine samples in Spain and Italy, and the presence of a Tn*10*-like-*tet*(B) transposon, the *sul3*-type I integron, and the *spvC* virulence gene of pSLT in these plasmids has already been shown [23]. Soon after, IncR plasmids were reported in monophasic isolates from human and pork samples in Portugal, and the term Southern European clone was then coined [25].

In the present study, six isolates of the Southern European clone were sequenced with short-read Illumina technology, and their IncR plasmids were reconstructed. The backbone of these plasmids was highly degenerated, only accounting for ca. 5 and 11 kb, in those of group 1 and group 2, respectively. However, it has incorporated large portions of exogenous DNA, mostly derived from pSLT and the IncI1-I(alpha) incompatibility group, but also from the IncN, IncFIB and IncP groups, although to a lesser extent and only present in some plasmids. Accordingly, the newly sequenced plasmids have a mosaic structure, comprising DNA from several sources as previously reported for pST1023 [21].

The IncR plasmids predominantly contributed to the MDR phenotype of the analyzed isolates. All resistance genes found in these plasmids were associated with intact or truncated genetic elements involved in DNA mobility, including insertion sequences, transposons, PCTs and integrons. Worth noting is the very high number of insertion sequences, belonging to different families, including IS*1*, IS*5*, IS*6*, IS*200*/IS*605* and IS*481*, with several of them, especially IS*26*, appearing in more than one copy.

Regarding integrons, transposons and PCTs, (i) the *sul3*-class 1 integron with the type I configuration (*intI1*/*dfrA12*-*orfF*-*aadA2*-*cmlA1*-*aadA1*-*qacH*-IS*440*-*sul3* [30]) was present in all plasmids. However, in two plasmids (pLSP 52/13 and pLSP 197/14, which are closely related), the *intI1* gene was truncated by the insertion of IS*26*. Those with the intact *intI1* gene were associated with a remnant Tn*21*, including the *tnpR* and *tnpA* genes, coding for the resolvase and transposase, respectively; (ii) the *tet*(B) gene was found as part of a defective Tn*10* in all but one plasmid that belonged to the tetracycline susceptible isolate LSP 40/13-1. In pLSP 40/12, this element is flanked by directly-oriented copies of IS*26*, forming a PCT, while in the remaining plasmids they are oppositely-oriented and also encompassed the *merRT* genes of a truncated *mer* locus; (iii) the *sil* genes were likewise embedded within a PCT in two of the plasmids (pLSP 40/12 and pLSP 40/13-1); (iv) the *strB*-Δ*strA* genes, detected in all IncR plasmids analyzed herein, were flanked by IS*Ec15* and IS*26*, the latter probably being responsible for the two different deletions affecting *strA*, which have left 117 and 184 out of the 267 nt of the intact gene in one and five plasmids, respectively. Finally, (v) one of the plasmids (pLSP 6/12) has acquired an additional DNA segment which contains a second class 1 integron, consisting only of *intI1*/*aadA22*, and the *erm*(B) gene.

Apart from diversification of the IncR plasmids, evolution of the Southern European clone can also occur through acquisition of additional plasmids. This was the case with LSP 40/13, in which a second resistance plasmid (pLSP 40/13-2) was found. This plasmid, of unknown incompatibility group, contains the *bla*_TEM-1B_ gene carried by the Tn*2* transposon and the *lnu*(G) gene. The latter gene was first identified in *Enterococcus faecalis*, embedded in transposon Tn*6260* [41], and then in many other Gram-positive and Gram-negative bacteria, carried by the chromosome or plasmids, and associated with different genetic elements. In *Enterobacterales*, IS*Pst2* is the main contributor to the spread of this gene [42]. In the present study, IS*Pst2*, which has *lnu*(G) integrated within the *tnp* gene, was acquired by pLSP 40/13-2 through replicative transposition, as demonstrated by the presence of the TSDs.

Three out of the six isolates investigated in the present study displayed the resistance pattern previously associated with the Southern European clone [20,21,23,25]. The remaining three showed variations due to acquisition of the *sil* genes conferring silver resistance and/or of the *erm*(B) and *lnu*(G) genes associated with resistance to antibiotics that prevent protein synthesis by binding to the 23S rRNA in the 50S ribosomal subunit [43,44]. These antibiotics, including macrolides and lincosamides, are widely used to fight infections caused by Gram-positive bacterial pathogens, and also by specific Gram-negative pathogens. However, classic macrolides, like erythromycin, are not considered for the treatment of infections produced by *Enterobacterales*, including *S. enterica*, due the intrinsic resistance associated with poor permeability of the outer membrane [43,45]. In contrast, azithromycin, a semisynthetic derivative of erythromycin, enters more readily and displays excellent potential against *Enterobacterales*. It thus became an antibiotic of choice to treat infections with MDR non-typhoid serovars of *S. enterica* in vulnerable patients as well as uncomplicated cases of enteric fever [43]. Although resistance of *S. enterica* to azithromycin remains low (for instance, 0.8% in Europe in 2020 [46]), several mechanisms preventing its action have already been reported. These include methylation of 23S rRNA by the products of *erm*(B) and *erm42*, modification of the antibiotic by the macrolide 2′-phosphotransferase encoded by *mph*(A), and enhanced expression of efflux pumps [47,48,49,50,51,52]. However, in the present study, the existence of *erm*(B) in one isolate of the Southern European monophasic clone did not correlate with azithromycin resistance. Similarly, several genes conferring resistance to lincosamides, including *erm*(B) and *lun*(G), have been reported in *S. enterica* [50,53]. However, as in the case of classic macrolides, this bacterium is intrinsically resistant to lincosamides, such as lincomycin and clindamycin [45], so resistance to these antibiotics was not tested for the *lnu*(G)-positive isolate detected herein.

The *erm*(B) and *lnu*(G) genes, both reported in the Southern European monophasic clone for the first time, are carried by mobile genetic elements located on plasmids. Thus, although *erm*(B) did not confer resistance to azithromycin, and the expected role of *lnu*(G) is masked by intrinsic resistance of *S. enterica* to lincosamides, this clone can act as a reservoir and contribute to the dispersion of these genes to other bacterial pathogens. In this respect, it is worth noting that IncR plasmids, including those found in the Southern European clone, are not conjugative. However, as shown in [21], they can be efficiently mobilized by plasmids of different incompatibility groups. It would be interesting to determine whether pLSP 40/13-2 is conjugative, and if so if it would be able to mobilize the co-resident IncR plasmid.

Apart from multiple resistance genes, pSLT-derived virulence genes were also provided by the IncR plasmids of the Southern European clone. In these plasmids, the pSLT DNA appeared as a contiguous segment (pLSP 40/12) or disrupted either by IS*26* (pLSP 40/13-1) or the *tet*(B)-containing element (in the remaining plasmids that belong to the second group). In all cases, the *spv* locus, which is the hallmark of serovar-specific virulence plasmids, remains intact. This locus strongly increases the ability of *Salmonella* to proliferate intracellularly during the extraintestinal phase of the disease, and has been associated with severe, disseminated infections in humans [54,55]. It comprises the *spvR* gene whose product is a transcriptional activator required for expression of the *spvABCD* operon. The products of the *spvB* and *spvC* genes are the main virulence factors provided by the serovar-specific virulence plasmids [56]. SpvB destabilizes the host cell cytoskeleton by covalently attaching an ADP-ribosyl group to G-actin monomers, preventing their polymerization. SpvC has phosphothreonine lyase activity and can inhibit MAP (mitogen-activated protein) kinase signaling, leading to down-regulation of cytokine release from infected cells. It is of note that in pST1023, but not in our plasmids, the *spcC* gene was disrupted by insertion of the *tet*(B)-containing element, which separates the pSLT DNA into two segments [21]. Another virulence gene of pSLT acquired by the IncR plasmids of the Southern European clone is *mig5*, which encodes a carbonic anhydrase that catalyses hydration of CO_2_ leading to HCO^3−^ formation and is expressed in *S*. Typhimurium after ingestion by macrophages [29].

As stated above, multiple copies of IS*26* are present in the IncR plasmids of the Southern European clone, and a single copy was also detected in the second resistance plasmid of one isolate (LSP 40/13). As suggested in [57], the eight bp TSDs and their patterns were used in the present study to track the replicative movement of IS*26*. This insertion sequence is playing a key role in the evolution and diversification of the IncR plasmids by (i) integration of individual copies at randomly selected target sites, without further rearrangements, as a result of intermolecular transposition; and by (ii) provoking deletions and inversions affecting the exogenous DNA associated with intramolecular transposition by the cis and trans pathways [57]. IS*26* could also have been transposing using the targeted conservative mechanism, which involves translocatable units (TU; consisting on one copy of IS*26* and the adjacent DNA) and leads to the generation of PCTs [27]. For instance, the additional segment of pLSP 40/13-1 where the *erm*(B) gene is located could have been acquired as a TU, now being bounded by directly-oriented copies of IS*26*. Many other PCTs were identified in the analyzed IncR plasmids, like those enclosing the *sil* region in pLSP 40/12 and pLSP 40/13-2; the *tet*(B) element in pLSP 40/12; and the *sul3*-type I integron of pLSP 52/13, pLSP 197/14 and pLSP 6/12. The *sil*- and the *tet*(B)-PCTs were previously reported in pST1023 [21]. The latter shares the IncR backbone with plasmids of the second group in the present study and carries the *sil* genes like those belonging to the first group, although they did not confer silver resistance [21]. Apart from IS*26*, many other insertion sequences have apparently been involved in the evolution of antibiotic resistance in the Southern European clone. A relevant example is the IS*Pst2*-mediated insertion of the *lnu*(G) gene into pLSP 40/13-2.

To establish the phylogenetic relationships between the isolates investigated in the present study, a phylogenetic tree was constructed including the genomes of ST1023 as well as of other isolates sharing the same large deletion removing *fljBA* but maintaining the *hin* gene. In the SNP-based phylogenetic tree, the Spanish isolates formed a coherent group together with the Italian isolate, all of them belonging to the Southern European clone. Other groups of closely related isolates were detected, but the large phylogenetic distances existing between them suggests that isolates having the *fljBA* deletion pattern previously associated with the Southern European and U.S./American clones, could comprise additional ones. Further phylogenetic studies are required to clarify this point.

## Figures and Tables

**Table 1:** Origin, general features and resistance properties of clinical isolates belonging to the Southern European clone of the monophasic variant of *Salmonella enterica* serovar Typhimurium.

Isolate ^a^	Patient: Sex ^b^/Age (Sample) ^c^ Hospital ^d^	Antigenic Formula	Phage Type ^e^	R-Profile	AMR-Phenotype/Genotype ^f^	R-Plasmid (Inc; Size in bp) ^g^ Other Plasmids (Size in bp)
LSP 64/15	F/56 (Fc) HFJ	4,5,12:i:-	RDNC	R1	CHL-STR-SUL-TET-TMP/ *cmlA1*, [*aadA1, aadA2, strA*-Δ1*, strB*]*, sul3, tet*(B), *dfrA12* *qacH*	**pLSP 64/15 (IncR; 126,138)**
LSP 197/14	F/1 (Fc) HUC	4,5,12:i:-	DT18	R1	CHL-STR-SUL-TET-TMP/ *cmlA1*, [*aadA1, aadA2, strA*-Δ2*, strB*], *sul3*, *tet*(B), *dfrA12* *qacH*	**pLSP 197/14 (IncR; 119,881)**
LSP 52/13	M/81 (Fc) HFJ	4,5,12:i:-	DT18	R1	CHL, STR, SUL, TET, TMP/ *cmlA1*, [*aadA1, aadA2, strA*-Δ2*, strB*], *sul3*, *tet*(B), *dfrA12* *qacH*	**pLSP52/13 (IncR; 113,363)**
LSP 40/13	F/33 (Fc) HUCA	4,12:i:-	DT120	R2	AMP, CHL, STR, SUL, TMP/ *cmlA1*, [*aadA1, aadA2, strA*-Δ2, *strB*], *sul3, dfrA12* *qacH, silPGABFCRSE*	**pLSP 40/13-1 (IncR; 121,068)**
					*bla*_TEM-1B_, *lnu*(G)	**pLSP 40/13-2 (ni; 33,942)**
					-	ni (4593); oriColE (3830); ni (3374)
LSP 40/12	F/53 (Fc) HUC	4,12:i:-	DT104	R3	CHL, STR, SUL, TET, TMP/ *cmlA1*, [*aadA1, aadA2, strA*-Δ2*, strB*]*, sul3, tet*(B), *dfrA12* *qacH, silPGABFCRSE*	**pLSP 40/12 (IncR; 124,546)**
					-	ni (4593); ni (4,066); ni (3374)
LSP 6/12	M/89 (B) HUCA	4,5,12:i:-	RDNC	R4	CHL, STR, SUL, TET, TMP/ *cmlA1*, [*aadA1, aadA2, aadA22*, *strA-*Δ2*, strB*], *sul3*, *tet*(B), *dfrA12, erm*(B) *qacH*	**pLSP 6/12 (IncR; 138,093)**

^a^, LSP, Laboratorio de Salud Pública, Asturias, Spain. ^b^, M, male; F, female. ^c^, Fc, feces; B, blood. ^d^, HUCA, Hospital Universitario Central de Asturias; HUC, Hospital Universitario de Cabueñes; HFJ, Hospital Fundación Jove. ^e^, RDNC, Reacted but Did Not Conform; DT, Definitive Type. ^f^, AMR, AntiMicrobial drug Resistance; AMP, ampicillin; CHL, chloramphenicol; STR, streptomycin; SUL, sulfonamides; TET, tetracycline; TMP, trimethoprim.; *strA*-Δ1, deleted gene consisting of 354 out of 804 nt; *strA*-Δ2, deleted gene consisting of 354 out of 804 nt. ^g^, Inc, Incompatibility group; Resistance plasmids are highlighted in bold; ni, not identified.

## Data Availability

The genome sequences generated in the present study were deposited at GenBank database under BioProject PRJNA911783, with accession numbers JAJCUM000000000, JAJCUL000000000, JAJCUK000000000, JAJCUJ000000000, JAJCUI000000000 and JAJCUH000000000, for LSP 6/12, LSP 40/12, LSP 43/13, LSP 52/13, LSP-197/14 and LSP 64/15, respectively.

## Supplementary Materials

The following supporting information can be downloaded at: https://www.mdpi.com/article/10.3390/antibiotics13040314/s1, Table S1: Accession numbers of genomes of monophasic isolates of *Salmonella enterica* belonging to the Southern European clone; parameters related to the quality of the assemblies; Table S2: Origin and accession numbers of the genomes of isolates of monophasic *Salmonella enterica* potentially belonging to the Southern European and U.S./American clones used for phylogenetic analysis in the present study; Table S3: Pairwise distance matrix calculated from SNP in the genomes of monophasic isolates of *Salmonella enterica*, potentially belonging to the Southern European and the U.S./American clones and derived from different sources and countries. References [58,59,60,61,62,63,64,65] are cited in the Supplementary Materials.

## References

[1] European Food Safety Authority (2023). The European Union One Health 2022 Zoonoses Report. EFSA J..

[2] Majowicz S.E., Musto J., Scallan E., Angulo F.J., Kirk M., O’Brien S.J., Jones T.F., Fazil A., Hoekstra R.M., International Collaboration on Enteric Disease “Burden of Illness” Studies (2010). The global burden of nontyphoidal *Salmonella* gastroenteritis. Clin. Infect. Dis..

[3] Issenhuth-Jeanjean S., Roggentin P., Mikoleit M., Guibourdenche M., de Pinna E., Nair S., Fields P.I., Weill F.X. (2014). Supplement 2008–2010 (no. 48) to the White-Kauffmann-Le Minor scheme. Res. Microbiol..

[4] Silverman M., Simon M. (1980). Phase variation: Genetic analysis of switching mutants. Cell.

[5] Dionisi A.M., Graziani C., Lucarelli C., Filetici E., Villa L., Owczarek S., Caprioli A., Luzzi I. (2009). Molecular characterization of multidrug-resistant strains of *Salmonella enterica* serotype Typhimurium and Monophasic variant (S. 4,[5],12:i:-) isolated from human infections in Italy. Foodborne Pathog. Dis..

[6] Echeita M.A., Herrera S., Usera M.A. (2001). Atypical, *fljB*-negative *Salmonella enterica* subsp. enterica strain of serovar 4,5,12:i:- appears to be a monophasic variant of serovar Typhimurium. J. Clin. Microbiol..

[7] Petrovska L., Mather A.E., AbuOun M., Branchu P., Harris S.R., Connor T., Hopkins K.L., Underwood A., Lettini A.A., Page A. (2016). Microevolution of monophasic *Salmonella* Typhimurium during epidemic, United Kingdom, 2005–2010. Emerg. Infect. Dis..

[8] Sun H., Wan Y., Du P., Bai L. (2020). The epidemiology of monophasic *Salmonella* Typhimurium. Foodborne Pathog. Dis..

[9] Switt A.I., Soyer Y., Warnick L.D., Wiedmann M. (2009). Emergence, distribution, and molecular and phenotypic characteristics of *Salmonella enterica* serotype 4,5,12:i. Foodborne Pathog. Dis..

[10] Zamperini K., Soni V., Waltman D., Sanchez S., Theriault E.C., Bray J., Maurer J.J. (2007). Molecular characterization reveals *Salmonella enterica* serovar 4,[5],12:i:- from poultry is a variant Typhimurium serovar. Avian Dis..

[11] Clark C.G., Landgraff C., Robertson J., Pollari F., Parker S., Nadon C., Gannon V.P.J., Johnson R., Nash J. (2020). Distribution of heavy metal resistance elements in Canadian *Salmonella* 4,[5],12:i:- populations and association with the monophasic genotypes and phenotype. PLoS ONE.

[12] Echeita M.A., Aladuena A., Cruchaga S., Usera M.A. (1999). Emergence and spread of an atypical *Salmonella enterica* subsp. *enterica* serotype 4,5,12:i:- strain in Spain. J. Clin. Microbiol..

[13] EFSA, EFSA Panel on Biological Hazards (BIOHAZ) (2010). Scientific Opinion on monitoring and assessment of the public health risk of “*Salmonella* Typhimurium-like” strains. EFSA J..

[14] Elnekave E., Hong S., Mather A.E., Boxrud D., Taylor A.J., Lappi V., Johnson T.J., Vannucci F., Davies P., Hedberg C. (2018). *Salmonella enterica* serotype 4,[5],12:i:- in swine in the United States Midwest: An emerging multidrug-resistant clade. Clin. Infect. Dis..

[15] Hopkins K.L., Kirchner M., Guerra B., Granier S.A., Lucarelli C., Porrero M.C., Jakubczak A., Threlfall E.J., Mevius D.J. (2010). Multiresistant *Salmonella enterica* serovar 4,[5],12:i:- in Europe: A new pandemic strain?. Euro Surveill..

[16] Soyer Y., Moreno Switt A., Davis M.A., Maurer J., McDonough P.L., Schoonmaker-Bopp D.J., Dumas N.B., Root T., Warnick L.D., Grohn Y.T. (2009). *Salmonella enterica* serotype 4,5,12:i:-, an emerging *Salmonella* serotype that represents multiple distinct clones. J. Clin. Microbiol..

[17] Arai N., Sekizuka T., Tamamura Y., Tanaka K., Barco L., Izumiya H., Kusumoto M., Hinenoya A., Yamasaki S., Iwata T. (2018). Phylogenetic characterization of *Salmonella enterica* serovar Typhimurium and its monophasic variant isolated from food animals in Japan revealed replacement of major epidemic clones in the last 4 decades. J. Clin. Microbiol..

[18] Agasan A., Kornblum J., Williams G., Pratt C.C., Fleckenstein P., Wong M., Ramon A. (2002). Profile of *Salmonella enterica* subsp. *enterica* (subspecies I) serotype 4,5,12:i:- strains causing food-borne infections in New York City. J. Clin. Microbiol..

[19] Ambrose S.J., Harmer C.J., Hall R.M. (2018). Compatibility and entry exclusion of IncA and IncC plasmids revisited: IncA and IncC plasmids are compatible. Plasmid.

[20] Arrieta-Gisasola A., Atxaerandio-Landa A., Garrido V., Grillo M.J., Martinez-Ballesteros I., Laorden L., Garaizar J., Bikandi J. (2020). Genotyping study of *Salmonella* 4,[5],12:i:- monophasic variant of serovar Typhimurium and characterization of the second-phase flagellar deletion by whole genome sequencing. Microorganisms.

[21] Calia C., Oliva M., Ferrara M., Minervini C.F., Scrascia M., Monno R., Mule G., Cumbo C., Marzella A., Pazzani C. (2022). Identification and characterisation of pST1023 a mosaic, multidrug-resistant and mobilisable IncR plasmid. Microorganisms.

[22] Garcia P., Guerra B., Bances M., Mendoza M.C., Rodicio M.R. (2011). IncA/C plasmids mediate antimicrobial resistance linked to virulence genes in the Spanish clone of the emerging *Salmonella enterica* serotype 4,[5],12:i. J. Antimicrob. Chemother..

[23] Garcia P., Hopkins K.L., Garcia V., Beutlich J., Mendoza M.C., Threlfall J., Mevius D., Helmuth R., Rodicio M.R., Guerra B. (2014). Diversity of plasmids encoding virulence and resistance functions in *Salmonella enterica* subsp. *enterica* serovar Typhimurium monophasic variant 4,[5],12:i:- strains circulating in Europe. PLoS ONE.

[24] Guerra B., Soto S.M., Arguelles J.M., Mendoza M.C. (2001). Multidrug resistance is mediated by large plasmids carrying a class 1 integron in the emergent *Salmonella enterica* serotype [4,5,12:i:-]. Antimicrob. Agents Chemother..

[25] Mourao J., Machado J., Novais C., Antunes P., Peixe L. (2014). Characterization of the emerging clinically-relevant multidrug-resistant *Salmonella enterica* serotype 4,[5],12:i:- (monophasic variant of *S.* Typhimurium) clones. Eur. J. Clin. Microbiol. Infect. Dis..

[26] Vazquez X., Garcia P., Garcia V., de Toro M., Ladero V., Heinisch J.J., Fernandez J., Rodicio R., Rodicio M.R. (2021). Genomic analysis and phylogenetic position of the complex IncC plasmid found in the Spanish monophasic clone of *Salmonella enterica* serovar Typhimurium. Sci. Rep..

[27] Harmer C.J., Pong C.H., Hall R.M. (2020). Structures bounded by directly-oriented members of the IS*26* family are pseudo-compound transposons. Plasmid.

[28] Nohmi T., Hakura A., Nakai Y., Watanabe M., Murayama S.Y., Sofuni T. (1991). *Salmonella typhimurium* has two homologous but different *umuDC* operons: Cloning of a new *umuDC*-like operon (*samAB*) present in a 60-megadalton cryptic plasmid of *S. typhimurium*. J. Bacteriol..

[29] Valdivia R.H., Falkow S. (1997). Fluorescence-based isolation of bacterial genes expressed within host cells. Science.

[30] Antunes P., Machado J., Peixe L. (2007). Dissemination of *sul3*-containing elements linked to class 1 integrons with an unusual 3′ conserved sequence region among *Salmonella* isolates. Antimicrob. Agents Chemother..

[31] Bolognese F., Di Lecce C., Galli E., Barbieri P. (1999). Activation and inactivation of *Pseudomonas stutzeri* methylbenzene catabolism pathways mediated by a transposable element. Appl. Environ. Microbiol..

[32] Garcia-Fernandez A., Fortini D., Veldman K., Mevius D., Carattoli A. (2009). Characterization of plasmids harbouring *qnrS1*, *qnrB2* and *qnrB19* genes in *Salmonella*. J. Antimicrob. Chemother..

[33] CLSI (2019). Performance Standards for Antimicrobial Susceptibility Testing.

[34] Vazquez X., Garcia-Fierro R., Fernandez J., Bances M., Herrero-Fresno A., Olsen J.E., Rodicio R., Ladero V., Garcia V., Rodicio M.R. (2023). Incidence and Genomic Background of Antibiotic Resistance in Food-Borne and Clinical Isolates of *Salmonella enterica* Serovar Derby from Spain. Antibiotics.

[35] Vielva L., de Toro M., Lanza V.F., de la Cruz F. (2017). PLACNETw: A web-based tool for plasmid reconstruction from bacterial genomes. Bioinformatics.

[36] Tatusova T., DiCuccio M., Badretdin A., Chetvernin V., Nawrocki E.P., Zaslavsky L., Lomsadze A., Pruitt K.D., Borodovsky M., Ostell J. (2016). NCBI prokaryotic genome annotation pipeline. Nucleic Acids Res..

[37] McClelland M., Sanderson K.E., Spieth J., Clifton S.W., Latreille P., Courtney L., Porwollik S., Ali J., Dante M., Du F. (2001). Complete genome sequence of *Salmonella enterica* serovar Typhimurium LT2. Nature.

[38] Kaas R.S., Leekitcharoenphon P., Aarestrup F.M., Lund O. (2014). Solving the problem of comparing whole bacterial genomes across different sequencing platforms. PLoS ONE.

[39] Felsenstein J. (1985). Confidence limits on phylogenies: An approach using the bootstrap. Evolution.

[40] Rodicio M.R., Herrero A., Rodríguez I., García P., Montero I., Beutlich J., Rodicio R., Guerra B., Mendoza M.C. (2011). Acquisition of antimicrobial resistance determinants by virulence plasmids specific for nontyphoid serovars of *Salmonella enterica*. Rev. Med. Microbiol..

[41] Zhu X.Q., Wang X.M., Li H., Shang Y.H., Pan Y.S., Wu C.M., Wang Y., Du X.D., Shen J.Z. (2017). Novel *lnu(G)* gene conferring resistance to lincomycin by nucleotidylation, located on Tn*6260* from *Enterococcus faecalis* E531. J. Antimicrob. Chemother..

[42] Li Y., Qiu Y., She J., Wang X., Dai X., Zhang L. (2021). Genomic characterization of a *Proteus* sp. strain of animal origin co-carrying *bla_NDM-1_* and *lnu(G)*. Antibiotics.

[43] Gomes C., Martinez-Puchol S., Palma N., Horna G., Ruiz-Roldan L., Pons M.J., Ruiz J. (2017). Macrolide resistance mechanisms in *Enterobacteriaceae*: Focus on azithromycin. Crit. Rev. Microbiol..

[44] Spizek J., Rezanka T. (2017). Lincosamides: Chemical structure, biosynthesis, mechanism of action, resistance, and applications. Biochem. Pharmacol..

[45] Stock I., Wiedemann B. (2000). Natural antibiotic susceptibility of *Salmonella enterica* strains. Int. J. Antimicrob. Agents.

[46] European Food Safety Authority, European Centre for Disease Prevention and Control (2022). The European Union Summary Report on Antimicrobial Resistance in zoonotic and indicator bacteria from humans, animals and food in 2019–2020. EFSA J..

[47] Chiou C.S., Hong Y.P., Wang Y.W., Chen B.H., Teng R.H., Song H.Y., Liao Y.S. (2023). Antimicrobial resistance and mechanisms of azithromycin resistance in nontyphoidal *Salmonella* isolates in Taiwan, 2017 to 2018. Microbiol. Spectr..

[48] Hong Y.P., Chen Y.T., Wang Y.W., Chen B.H., Teng R.H., Chen Y.S., Chiou C.S. (2023). Integrative and conjugative element-mediated azithromycin resistance in multidrug-resistant *Salmonella enterica* serovar Albany. Antimicrob. Agents Chemother..

[49] Hooda Y., Sajib M.S.I., Rahman H., Luby S.P., Bondy-Denomy J., Santosham M., Andrews J.R., Saha S.K., Saha S. (2019). Molecular mechanism of azithromycin resistance among typhoidal *Salmonella* strains in Bangladesh identified through passive pediatric surveillance. PLoS Negl. Trop. Dis..

[50] Rodrigues G.L., Panzenhagen P., Ferrari R.G., Dos Santos A., Paschoalin V.M.F., Conte-Junior C.A. (2020). Frequency of antimicrobial resistance genes in *Salmonella* from Brazil by in silico whole-genome sequencing analysis: An overview of the last four decades. Front. Microbiol..

[51] Wang J., Li Y., Xu X., Liang B., Wu F., Yang X., Ma Q., Yang C., Hu X., Liu H. (2017). Antimicrobial resistance of *Salmonella enterica* serovar Typhimurium in Shanghai, China. Front. Microbiol..

[52] Wang M., Tran J.H., Jacoby G.A., Zhang Y., Wang F., Hooper D.C. (2003). Plasmid-mediated quinolone resistance in clinical isolates of *Escherichia coli* from Shanghai, China. Antimicrob. Agents Chemother..

[53] Zhang Z., Tian X., Shi C. (2022). Global spread of MCR-producing *Salmonella enterica* isolates. Antibiotics.

[54] Fierer J. (2001). Extra-intestinal *Salmonella* infections: The significance of *spv* genes. Clin. Infect. Dis..

[55] Fierer J., Krause M., Tauxe R., Guiney D. (1992). *Salmonella typhimurium* bacteremia: Association with the virulence plasmid. J. Infect. Dis..

[56] Guiney D.G., Fierer J. (2011). The role of the *spv* genes in *Salmonella* pathogenesis. Front. Microbiol..

[57] He S., Hickman A.B., Varani A.M., Siguier P., Chandler M., Dekker J.P., Dyda F. (2015). Insertion sequence IS*26* reorganizes plasmids in clinically isolated multidrug-resistant bacteria by replicative transposition. mBio.

[58] Leeper M.M., Tolar B.M., Griswold T., Vidyaprakash E., Hise K.B., Williams G.M., Im S.B., Chen J.C., Pouseele H., Carleton H.A. (2023). Evaluation of whole and core genome multilocus sequence typing allele schemes for *Salmonella enterica* outbreak detection in a national surveillance network, PulseNet USA. Front. Microbiol..

[59] Ji H.J., Jang A.Y., Song J.Y., Ahn K.B., Han S.H., Bang S.J., Jung H.K., Hur J., Seo H.S. (2022). Development of live attenuated *Salmonella* Typhimurium vaccine strain using radiation mutation enhancement technology (R-MET). Front. Immunol..

[60] Maguire M., Khan A.S., Adesiyun A.A., Georges K., Gonzalez-Escalona N. (2022). Genomic comparison of eight closed genomes of multidrug-resistant *Salmonella enterica* strains isolated from broiler farms and processing plants in Trinidad and Tobago. Front. Microbiol..

[61] Li C., Tyson G.H., Hsu C.H., Harrison L., Strain E., Tran T.T., Tillman G.E., Dessai U., McDermott P.F., Zhao S. (2021). Long-Read Sequencing Reveals Evolution and Acquisition of Antimicrobial Resistance and Virulence Genes in *Salmonella enterica*. Front. Microbiol..

[62] Ge B., Mukherjee S., Li C., Harrison L.B., Hsu C.H., Tran T.T., Whichard J.M., Dessai U., Singh R., Gilbert J.M. (2024). Genomic analysis of azithromycin-resistant *Salmonella* from food animals at slaughter and processing, and retail meats, 2011–2021, United States. Microbiol. Spectr..

[63] Timme R.E., Lafon P.C., Balkey M., Adams J.K., Wagner D., Carleton H., Strain E., Hoffmann M., Sabol A., Rand H. (2020). Gen-FS coordinated proficiency test data for genomic foodborne pathogen surveillance, 2017 and 2018 exercises. Sci. Data.

[64] Chen Z., Kuang D., Xu X., Gonzalez-Escalona N., Erickson D.L., Brown E., Meng J. (2020). Genomic analyses of multidrug-resistant *Salmonella* Indiana, Typhimurium, and Enteritidis isolates using MinION and MiSeq sequencing technologies. PLoS ONE.

[65] Nguyen S.V., Harhay D.M., Bono J.L., Smith T.P., Fields P.I., Dinsmore B.A., Santovenia M., Kelley C.M., Wang R., Bosilevac J.M. (2016). Complete and Closed Genome Sequences of 10 *Salmonella enterica* subsp. *enterica* Serovar Anatum Isolates from Human and Bovine Sources. Genome Announc..

