# Supplementary material for: Insights into the Evolution of IncR Plasmids Found in the Southern European Clone of the Monophasic Variant of Salmonella enterica Serovar Typhimurium
Source: Antibiotics (Basel). 2024 Mar 29;13(4):314. doi: 10.3390/antibiotics13040314 (PMC11047700; doi:10.3390/antibiotics13040314)
Supplement: Supplementary file 1 [file antibiotics-13-00314-s001.zip › antibiotics-2924064-supplementary.pdf]

antibiotics MDPI

Supplementary material

Insights into the evolution of IncR plasmids found in the Southern European clone of the monophasic variant of *Salmonella enterica* serovar Typhimurium

Table S1. Accession numbers of genomes of monophasic isolates of *Salmonella enterica* belonging to the Southern European clone; parameters related to the quality of the assemblies

Isolate[a]	Kmer	Contigs	N50	Longest contig (bp)	Total bp in contigs	Contigs > 1 kb	GenBank accession number
LSP 6/12	131	112	260872	930160	930160	53	JAJCUM000000000
LSP 40/12	89	120	324386	929430	4963851	49	JAJCUL000000000
LSP 40/13	131	188	324728	478577	5061947	78	JAJCUK000000000
LSP 52/13	131	189	324746	929941	4973925	56	JAJCUJ000000000
LSP 197/14	131	198	324887	930230	4963683	51	JAJCUI000000000
LSP 64/15	131	103	260762	929941	4920808	45	JAJCUH000000000

[a], Isolates are designated with the initials of the center which supplied them (Laboratorio de Salud Pública, Principality of Asturias, Spain), followed by a serial number/last two numbers of the year of recovery.

Table S2. Origin and accession numbers of the genomes of isolates of monophasic *Salmonella enterica* potentially belonging to the Southern European and U.S./American clones used for phylogenetic analysis in the present study

Isolate	Size (bp)	Source	Country	Isolation Year	GenBank accession No.	Reference
08-1736	4,822,189	-	-	-	CP006602.1	-
2011K-0052	4,857,411	Human	USA	2011	CP114540.1	[58]
ATOMSal-L6	4,784,174	Swine	South Korea	2019	CP098438.1	[59]
CFSAN057258	4,820,861	Water	USA	2016	CP076086.1	-
CFSAN103852	4,836,001	Environmental	Trinidad and Tobago	2016	CP066328.1	[60]
CVM N18S0993	4,817,368	Ground turkey	USA	2018	CP082571.1	[61]
FDAARGOS_768	4,857,490	-	-	-	CP041005.1	-
FDAARGOS_878	4,874,787	-	-		CP065718.1	-
FELIX_MS205	4,857,968	-	-	-	CP139746.1	-
FSIS12032448	4,861,061	Animal-Calf-Bob Veal	USA	2020	CP123684.1	[62]
HJL222	4,823,435	Swine	South Korea	2015	CP098741.1	[59]
PNCS000211	4,789,195	Human	Canada	2010	CP039716.1	-
PNCS007087	4,787,290	Human	Canada	2010	CP044957.1	-
PNCS007098	4,773,032	Human	Canada	2011	CP044967.1	
PNCS014846	4,783,087	Human	Canada	2008	CP039558.1	
PNCS014849	4,815,188	Human	Canada	2008	CP039565.1	
PNCS014850	4,815,492	Human	Canada	2008	CP039567.1	
PNCS014853	4,815,118	Human	Canada	2009	CP039713.1	
PNCS014854	4,817,234	Human	Canada	2009	CP037874.1	
PNCS014859	4,814,945	Human	Canada	2010	CP039585.1	
PNCS015054	4,786,287	Human	Canada	2010	CP037877.1	
SAP18-6199	4,813,658	-	USA	2018	CP040900.1	[63]
ST56	4,948,999	Human	China	2011	CP050739.1	[64]
ST90	4,906,732	Duck	China	2012	CP050734.1	[64]

USDA-ARS-USMARC-1880	4,815,208	Bovine carcass	USA	2003	CP014981.1	[65]
ST1023	5,169,410	Human	Italy	2008	JANKYD000000000	[21]
LSP 6/12	4,967,484	Human	Spain	2012	JAJCUM000000000	This study
LSP 40/12	4,966,702	Human	Spain	2012	JAJCUL000000000	This study
LSP 43/13	5,056,065	Human	Spain	2013	JAJCUK000000000	This study
LSP 52/13	4,975,638	Human	Spain	2013	JAJCUJ000000000	This study
LSP 197/14	4,960,478	Human	Spain	2014	JAJCUI000000000	This study
LSP 64/15	4,921,913	Human	Spain	2015	JAJCUH000000000	This study

–, information not available.

References

21. Calia, C.; Oliva, M.; Ferrara, M.; Minervini, C.F.; Scrascia, M.; Monno, R.; Mule, G.; Cumbo, C.; Marzella, A.; Pazzani, C. Identification and characterisation of pST1023 a mosaic, multidrug-resistant and mobilisable IncR plasmid. *Microorganisms* **2022**, *10*, doi:10.3390/microorganisms10081592.

58. Leeper, M.M.; Tolar, B.M.; Griswold, T.; Vidyaprakash, E.; Hise, K.B.; Williams, G.M.; Im, S.B.; Chen, J.C.; Pouseele, H.; Carleton, H.A. Evaluation of whole and core genome multilocus sequence typing allele schemes for *Salmonella enterica* outbreak detection in a national surveillance network, PulseNet USA. *Front Microbiol* **2023**, *14*, 1254777, doi:10.3389/fmicb.2023.1254777.

59. Ji, H.J.; Jang, A.Y.; Song, J.Y.; Ahn, K.B.; Han, S.H.; Bang, S.J.; Jung, H.K.; Hur, J.; Seo, H.S. Development of live attenuated *Salmonella* Typhimurium vaccine strain using radiation mutation enhancement technology (R-MET). *Front Immunol* **2022**, *13*, 931052, doi:10.3389/fimmu.2022.931052.

60. Maguire, M.; Khan, A.S.; Adesiyun, A.A.; Georges, K.; Gonzalez-Escalona, N. Genomic comparison of eight closed genomes of multidrug-resistant *Salmonella enterica* strains isolated from broiler farms and processing plants in Trinidad and Tobago. *Front Microbiol* **2022**, *13*, 863104, doi:10.3389/fmicb.2022.863104.

61. Li, C.; Tyson, G.H.; Hsu, C.H.; Harrison, L.; Strain, E.; Tran, T.T.; Tillman, G.E.; Dessai, U.; McDermott, P.F.; Zhao, S. Long-Read Sequencing Reveals Evolution and Acquisition of Antimicrobial Resistance and Virulence Genes in Salmonella enterica. *Front Microbiol* **2021**, *12*, 777817, doi:10.3389/fmicb.2021.777817.

62. Ge, B.; Mukherjee, S.; Li, C.; Harrison, L.B.; Hsu, C.H.; Tran, T.T.; Whichard, J.M.; Dessai, U.; Singh, R.; Gilbert, J.M.; et al. Genomic analysis of azithromycin-resistant *Salmonella* from food animals at slaughter and processing, and retail meats, 2011-2021, United States. *Microbiol Spectr* **2024**, *12*, e0348523, doi:10.1128/spectrum.03485-23.

63. Timme, R.E.; Lafon, P.C.; Balkey, M.; Adams, J.K.; Wagner, D.; Carleton, H.; Strain, E.; Hoffmann, M.; Sabol, A.; Rand, H.; et al. Gen-FS coordinated proficiency test data for genomic foodborne pathogen surveillance, 2017 and 2018 exercises. *Sci Data* **2020**, *7*, 402, doi:10.1038/s41597-020-00740-7.

64. Chen, Z.; Kuang, D.; Xu, X.; Gonzalez-Escalona, N.; Erickson, D.L.; Brown, E.; Meng, J. Genomic analyses of multidrug-resistant *Salmonella* Indiana, Typhimurium, and Enteritidis isolates using MinION and MiSeq sequencing technologies. *PLoS One* **2020**, *15*, e0235641, doi:10.1371/journal.pone.0235641.

65. Nguyen, S.V.; Harhay, D.M.; Bono, J.L.; Smith, T.P.; Fields, P.I.; Dinsmore, B.A.; Santovenia, M.; Kelley, C.M.; Wang, R.; Bosilevac, J.M.; et al. Complete and Closed Genome Sequences of 10 *Salmonella enterica* subsp. *enterica* Serovar Anatum Isolates from Human and Bovine Sources. *Genome Announc* **2016**, *4*, doi:10.1128/genomeA.00447-16.

antibiotics

MDPI

Table S3. Pairwise distance matrix calculated from SNP in the genomes of monophasic isolates of *Salmonella enterica,* potentially belonging to the Southern European and the U.S./American clones and derived from different sources and countries.

	08-1736	2011K-0052	ATOMSal-L6	CFSAN057258	CFSAN103852	CVM_N18S0993	FDAARGOS_768	FDAARGOS_878	FELIX_MS205	FSIS12032448	HJL222	LSP 197/14	LSP 40/12	LSP 40/13	LSP 52/13	LSP 64/15	LSP 6/12	PNCS000211	PNCS007087	PNCS007098	PNCS014846	PNCS014849	PNCS014850	PNCS014853	PNCS014854	PNCS014859	PNCS015054	SAP18-6199	ST56	ST90	USDA-ARS-USMARC-1880	ST1023
**08-1736**	0	53	663	574	47	61	536	550	546	576	655	758	760	769	758	766	755	60	66	65	57	46	63	49	49	756	58	735	653	647	579	755
**2011K-0052**	53	0	674	585	58	72	547	561	557	587	666	763	767	776	763	771	760	71	77	76	68	57	74	60	60	765	69	744	664	658	590	762
**ATOMSal-L6**	663	674	0	465	668	670	155	167	163	467	8	655	657	666	655	663	652	669	675	672	666	655	666	658	666	657	667	636	249	243	510	652
**CFSAN057258**	574	585	465	0	579	581	338	352	348	106	457	608	610	619	608	616	605	580	586	583	577	566	577	569	579	602	578	577	455	449	119	605
**CFSAN103852**	47	58	668	579	0	66	541	555	551	581	660	761	763	772	761	769	758	65	71	70	62	51	68	54	54	761	63	740	658	652	584	758
**CVM_N18S0993**	61	72	670	581	66	0	543	557	553	583	662	763	765	774	763	771	760	61	67	66	58	15	60	18	68	763	59	742	660	654	586	760
**FDAARGOS_768**	536	547	155	338	541	543	0	14	10	340	147	530	532	541	530	538	527	542	548	545	539	528	539	531	539	530	540	509	145	139	383	527
**FDAARGOS_878**	550	561	167	352	555	557	14	0	20	354	159	540	542	551	540	548	537	556	562	559	553	542	553	545	553	542	554	521	157	151	397	537
**FELIX_MS205**	546	557	163	348	551	553	10	20	0	350	155	540	542	551	540	548	537	552	558	555	549	538	549	541	549	540	550	519	153	147	393	537
**FSIS12032448**	576	587	467	106	581	583	340	354	350	0	459	598	600	609	598	606	595	582	588	585	579	568	579	571	581	598	580	573	457	451	151	595
**HJL222**	655	666	8	457	660	662	147	159	155	459	0	647	649	658	647	655	644	661	667	664	658	647	658	650	658	649	659	628	241	235	502	644
**LSP 197/14**	758	763	655	608	761	763	530	540	540	598	647	0	52	61	0	34	5	762	768	765	759	748	759	751	761	514	760	513	643	637	567	45
**LSP 40/12**	760	767	657	610	763	765	532	542	542	600	649	52	0	19	52	60	49	764	770	767	761	750	761	753	763	518	762	517	645	639	569	39
**LSP 40/13**	769	776	666	619	772	774	541	551	551	609	658	61	19	0	61	69	58	773	779	776	770	759	770	762	772	527	771	526	654	648	578	48
**LSP 52/13**	758	763	655	608	761	763	530	540	540	598	647	0	52	61	0	34	5	762	768	765	759	748	759	751	761	514	760	513	643	637	567	45
**LSP 64/15**	766	771	663	616	769	771	538	548	548	606	655	34	60	69	34	0	31	770	776	773	767	756	767	759	769	522	768	521	651	645	575	53
**LSP 6/12**	755	760	652	605	758	760	527	537	537	595	644	5	49	58	5	31	0	759	765	762	756	745	756	748	758	511	757	510	640	634	564	42
**PNCS000211**	60	71	669	580	65	61	542	556	552	582	661	762	764	773	762	770	759	0	16	15	7	46	63	49	67	762	8	741	659	653	585	759
**PNCS007087**	66	77	675	586	71	67	548	562	558	588	667	768	770	779	768	776	765	16	0	19	11	52	69	55	73	768	12	747	665	659	591	765
**PNCS007098**	65	76	672	583	70	66	545	559	555	585	664	765	767	776	765	773	762	15	19	0	10	51	68	54	72	765	9	744	662	656	588	762
**PNCS014846**	57	68	666	577	62	58	539	553	549	579	658	759	761	770	759	767	756	7	11	10	0	43	58	46	64	759	3	738	656	650	582	756
**PNCS014849**	46	57	655	566	51	15	528	542	538	568	647	748	750	759	748	756	745	46	52	51	43	0	45	3	53	748	44	727	645	639	571	745
**PNCS014850**	63	74	666	577	68	60	539	553	549	579	658	759	761	770	759	767	756	63	69	68	58	45	0	48	68	759	61	738	656	650	582	756
**PNCS014853**	49	60	658	569	54	18	531	545	541	571	650	751	753	762	751	759	748	49	55	54	46	3	48	0	56	751	47	730	648	642	574	748
**PNCS014854**	49	60	666	579	54	68	539	553	549	581	658	761	763	772	761	769	758	67	73	72	64	53	68	56	0	759	65	738	656	650	584	758
**PNCS014859**	756	765	657	602	761	763	530	542	540	598	649	514	518	527	514	522	511	762	768	765	759	748	759	751	759	0	760	512	647	641	561	511
**PNCS015054**	58	69	667	578	63	59	540	554	550	580	659	760	762	771	760	768	757	8	12	9	3	44	61	47	65	760	0	739	657	651	583	757
**SAP18-6199**	735	744	636	577	740	742	509	521	519	573	628	513	517	526	513	521	510	741	747	744	738	727	738	730	738	512	739	0	626	620	538	512
**ST56**	653	664	249	455	658	660	145	157	153	457	241	643	645	654	643	651	640	659	665	662	656	645	656	648	656	647	657	626	0	66	500	640
**ST90**	647	658	243	449	652	654	139	151	147	451	235	637	639	648	637	645	634	653	659	656	650	639	650	642	650	641	651	620	66	0	494	634
**USDA-ARS-USMA**	579	590	510	119	584	586	383	397	393	151	502	567	569	578	567	575	564	585	591	588	582	571	582	574	584	561	583	538	500	494	0	564
**ST1023**	755	762	652	605	758	760	527	537	537	595	644	45	39	48	45	53	42	759	765	762	756	745	756	748	758	511	757	512	640	634	564	0

Min:0; max:779
